# Age-related mortality in 61,993 confirmed COVID-19 cases over three epidemic waves in Aragon, Spain. Implications for vaccination programmes

**DOI:** 10.1371/journal.pone.0261061

**Published:** 2021-12-09

**Authors:** Diego Casas-Deza, Vanesa Bernal-Monterde, Angel Nicolás Aranda-Alonso, Enrique Montil-Miguel, Ana Belen Julián-Gomara, Laura Letona-Giménez, Jose M. Arbones-Mainar

**Affiliations:** 1 Gastroenterology Department, Miguel Servet University Hospital, Zaragoza, Spain; 2 Instituto de Investigación Sanitaria (IIS) Aragon, Zaragoza, Spain; 3 Internal Medicine Department, Miguel Servet University Hospital, Zaragoza, Spain; 4 Translational Research Unit, Miguel Servet University Hospital, Instituto Aragonés de Ciencias de la Salud, Zaragoza, Spain; 5 Centro de Investigación Biomédica en Red Fisiopatología Obesidad y Nutrición (CIBERObn), Instituto Salud Carlos III, Madrid, Spain; Azienda Ospedaliero Universitaria Careggi, ITALY

## Abstract

**Background:**

Risk for severe COVID-19 increases with age. Different vaccination strategies are currently being considered, including those aimed at slowing down transmission and those aimed at providing direct protection to those most at risk.

**Methods:**

The objectives of the current study were i) to assess age-related incidence and survival between PCR-diagnosed COVID-19 cases (n = 61,993) in the Autonomous Community of Aragon from March to November 2020, and ii) to characterize age differences regarding the course of the disease in hospitalized patients in a tertiary university hospital.

**Results:**

We found a similar incidence of COVID-19 in individuals between 10 and 79 years. Incidence increased in those over 80 years possibly because of the elevated transmission within the nursing homes. We observed a profound disparity among age groups; case fatality rates (CFRs) were near 0 in cases younger than 39 years throughout different waves. In contrast, there was an age-dependent and progressive increase in the CFRs, especially during the first pandemic wave. SARS-CoV-2 infection caused a more severe and rapid progression in older patients. The elderly required faster hospitalization, presented more serious symptoms on admission, and had a worse clinical course. Hospitalized older individuals, even without comorbidities, had an increased mortality risk directly associated with their age. Lastly, the existence of comorbidities dramatically increased the CFRs in the elderly, especially in males.

**Conclusion:**

The elevated incidence of COVID-19 and the vulnerability of the elderly call for their prioritization in vaccination and targeted prevention measures specifically focused on this aged population.

## Introduction

In many countries, the 2020 COVID-19 epidemic pattern had a catastrophic first wave during the spring, with a surge in the summer and a larger third wave in the fall [1]. Based on different real-world series, the case-fatality rate (CFR) of the elderly and very elderly patients is much higher than the overall [2–5]. Paradoxically, there are few studies focused on this population, especially about very older patients (>75–80 years-old) [5–9]. Age is associated with increased prevalence of comorbidities, polypharmacy, cognitive impairment, dependence, and increased frailty [10]. Also, immuno-senescence may influence a different response of the patient against infections [11].

To understand the age-dependent mechanism of mortality from COVID-19, we must be able to accurately estimate the fatality rate according to the different age ranges. This requires the implementation of active epidemiological surveillance capable of detecting infections in real time and provides granular data to better characterize the clinical course of the disease, especially in those groups with higher risk of developing a severe illness [12].

The appearance of new methods and technologies for data integration and analysis opens the possibility of evaluating information currently generated by different information systems, repositories and other sources of data. These new methods combine integration tools (extraction, loading and transformation of data) and tools for data analysis to generate new scientific evidence based on real-world data [13, 14].

The objective of the current study was i) to assess age-related incidence and survival in polymerase chain reaction (PCR)-diagnosed COVID-19 cases in the Autonomous Community of Aragon systematically collected by the BIGAN registry from March to November 2020, and ii) to characterize the course of COVID-19 in older population hospitalized in a tertiary university hospital.

## Material and methods

The case definition includes any individual with laboratory confirmation of SARS-CoV-2 infection by real time (RT)-PCR test, irrespective of clinical signs and symptoms.

### Data sources

Epidemiological data from the BIGAN database were analyzed. BIGAN is coordinated by the Institute of Health Sciences of Aragon and collects data from different institutions (Emergency departments, Primary Care department, Public Health department, Electronic Prescription and Hospital Information System and Basic Minimum Basic Data Set on Hospital Discharge) from the Autonomous Community of Aragon (Spain) to be included in a single database. Data collected on all laboratory-confirmed cases included information on sex, age, birthplace, clinical severity, date of symptoms onset, date of diagnosis, date of hospitalization, and clinical outcome. This study was approved by the Research Ethics Committee of the Autonomous Community of Aragon: CEIC-A (C.P.—C.I. PI20/366). All data were fully anonymized before accessing them and our research ethics committee waived the requirement for informed consent.

The clinical course of the patients affected with COVID-19 was investigated in all consecutive patients that were PCR-diagnosed with SARS-CoV-2 and hospitalized at University Hospital Miguel Servet (Zaragoza, Aragon, Spain) between February 25, 2020 and April 8, 2020. We excluded a 1-year-old infant and 2 pregnant women because we were interested in the association of comorbidities and severity of COVID-19 and the Charlson Comorbidity Index was not developed for pediatric or obstetric populations [15]. Clinical data were manually extracted from patients´ electronic medical records. Biochemical tests and drug therapy during hospitalization were directly pulled out from the laboratory and hospital pharmacy data management systems respectively. Data curation was conducted by the Translational Research Unit of our hospital [16] after obtaining the approval from the Research Ethics Committee of the Autonomous Community of Aragon: CEIC-A (C.P.—C.I. EPA20/023) which authorized waived informed consent. Comorbidity burden was measured using the Charlson Comorbidity Index [15]. Acute Respiratory Distress Syndrome (ARDS), chronic kidney disease, sepsis, and multi-organ failure were defined as previously described [17–19]. Diagnostic and Statistical Manual of Mental Disorders (DSM)-5 criteria were used for the diagnosis of dementia. Patients were considered to have high blood pressure, diabetes mellitus or dyslipidemia if they had a prior clinical diagnosis or have been on pharmacological treatment for these conditions.

Radiological findings at admission were specifically reviewed in every patient by two expert radiologists, blinded to patient’s age. The severity was evaluated according to the Spanish Society of Radiology recommendations using the Radiological Index for admission consideration (0–2: mild; 3–5: moderate; 6–8: severe). This scale is based on the number of injuries, distribution, laterality, and the presence of pleural effusion and/or consolidation [20].

### Data analysis

Descriptive visual analyses were used to illustrate incidence and mortality rates stratified by age group and sex. Case fatality rates (CFR) were calculated as the total number of COVID-19 deaths divided by the total number of cases.

Hospitalization data were summarized the data as median (interquartile range [IQR]), mean (standard deviation) or percentages. Kruskal-Wallis and χ2 test with Yates correction for continuity were used for comparing continuous and categorical variables respectively. Tests of significance were two-tailed. Pairwise comparisons were adjusted for multiple testing using the Benjamini & Hochberg method [21]. Analyses were performed using R statistical software (version 4.0.3, https://www.r-project.org/) and the lubridate [22], compareGroups [23], and ggplot2 [24] packages.

## Results

### Epidemiological characteristics of COVID-19 cases at the regional level

**Fig 1:**
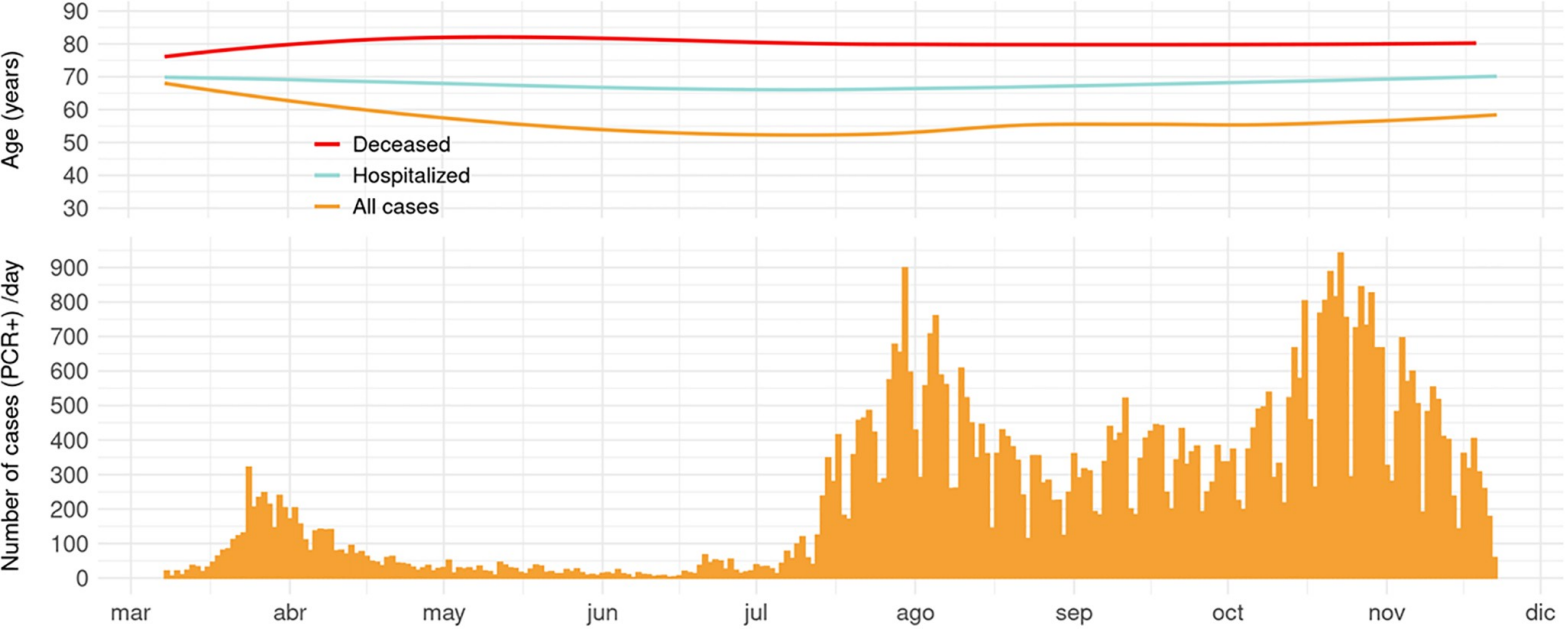
Daily confirmed COVID-19 cases (n = 61,993) and smoothed trends for the median age of all the cases, hospitalized cases, and deceased cases.

We included 61,993 PCR-diagnosed cases of COVID-19 (46.6% women) in Aragon (population = 1.32 mil) from March 8 to November 22, 2020. Here, we summarized the daily incidence together with the median age for all the confirmed cases, as well as hospitalized and deceased cases (Fig 1). Following the detection of the initial cases imported from China into Europe during spring, there was a summer wave of transmission likely originated by temporary foreign farm workers (S1 Fig). Cases began to spike again in October synchronously with another wave that also affected most Spanish regions and other European countries. The median age of confirmed COVID-19 cases varied widely from 71 in April to 37.9 years in July and back to 48.6 in November. Some variation was also observed in the median age for hospitalized individuals, ranging between 80.5 (April) and 67 years (July). In contrast, the median age for the deceased individuals remained fairly constant between 89.1 and 85.5 years and 99% of deceased were over 50 years.

**Fig 2:**
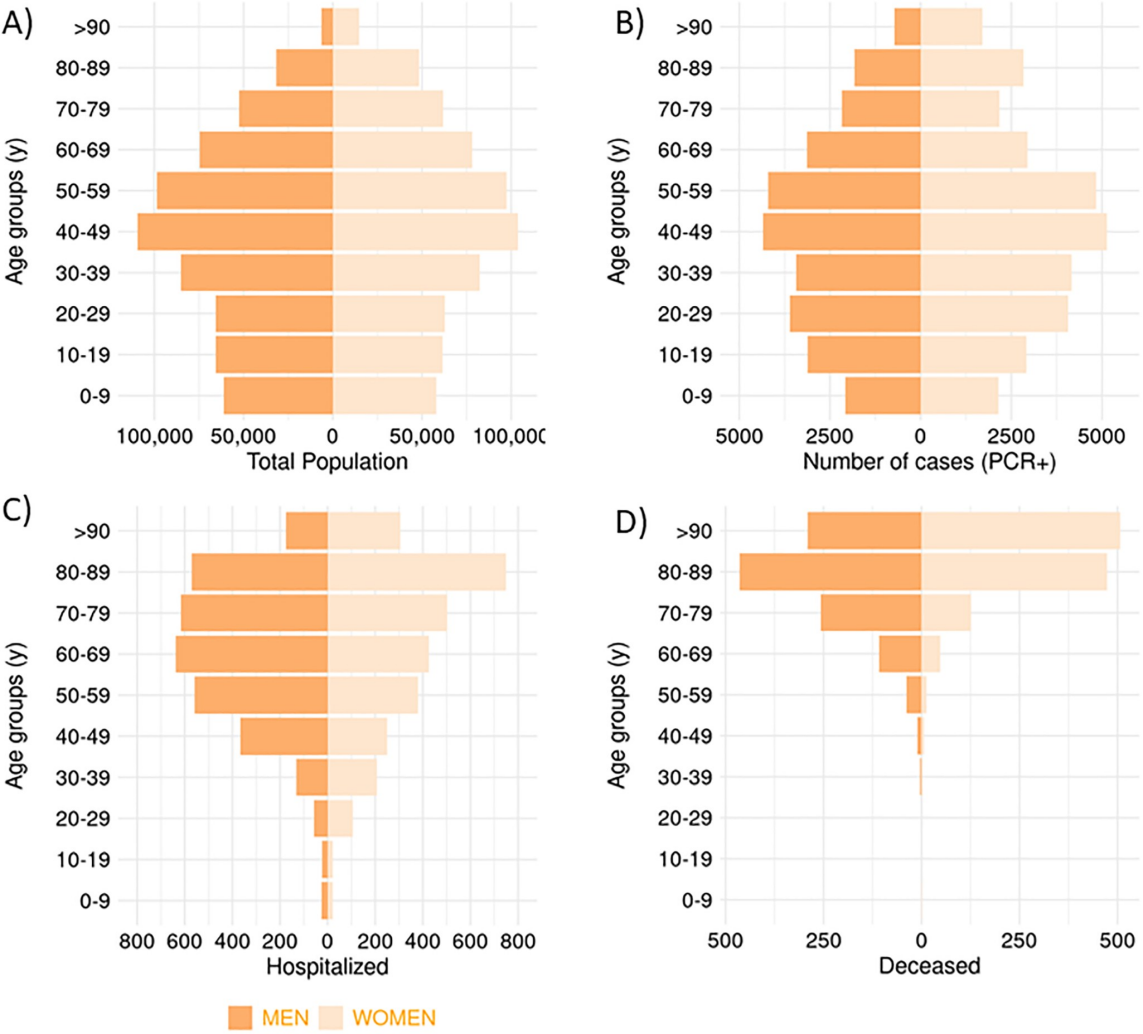
Population pyramid of Aragón (A) and number of PCR+ cases (B), hospitalized (C), and deceased (D) since March 3 to November 22 in each age group.

Aragón demographics shows the highest number of people in the 40–49 years age group (16.2% of the population) and a predominantly aged population in which 42.7% of the population is above the age of 50 and 11.4% is over 75 years (Fig 2A). Similar percentages were observed in the incidence of COVID-19 cases. The larger number of cases was observed among those in the 40–49 years range, 43.2% of the cases were older than 50 years, and 14.9% were over 75 years (Fig 2B). However, when examining the severity of the disease in terms of hospitalization and mortality rates by age group, older adults (>75 y) accounted for 46.4% of hospitalizations and 84.4% of the total number of deaths (Fig 2C and 2D).

**Fig 3:**
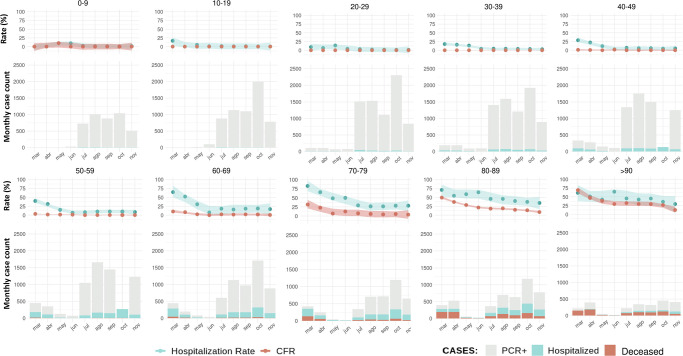
Hospitalization rate and case fatality rate (CFR) over time and by age groups for individuals with positive PCR for COVID-19 in Aragón. Figures are based on events registered in the month. Case-to-event rates are calculated as hospitalizations (HR) and deaths (CFR) divided by cases in the same period. Uncertainty bands represent the 95% confidence interval of the local polynomial regression (“loess”).

When changes in both hospitalization rate and CFR were studied longitudinally, we found a profound disparity among age groups with CFRs near 0 in cases younger than 60 years (Fig 3). Those negligible values remained constant over time and regardless of the incidence changes that occurred during the follow-up within the age group. In contrast, there was an age-dependent and progressive increase in the CFRs, especially during the initial months of the pandemic. Notably, from May onwards the average CFR among COVID-19 cases was 1.69 in the 60–69 age range, 5.19 in the 70–79 age group, and increased markedly among those over 80 years (CFR = 18.13). Again, CFRs among the elderly were independent of the monthly variation of incidence for each age range.

**Fig 4:**
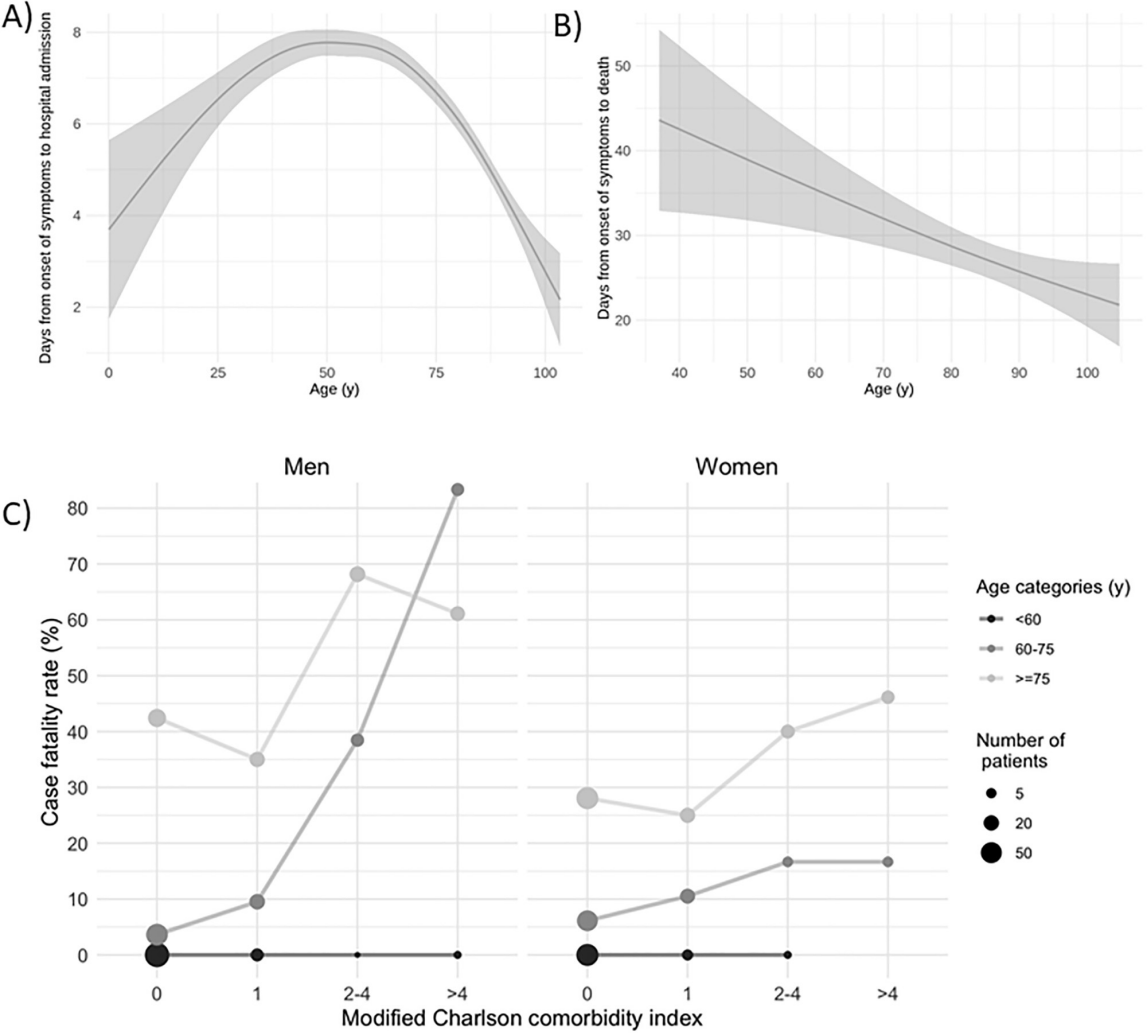
Symptom onset to event according to age and case fatality rate according to sex, age and comorbidities.

Hospitalization rate also decreased during the second and third waves for all age groups (Fig 3). The median number of days from the onset of COVID-19 symptoms to hospital admission was 6 (IQR: 6–9). We found that the average time from illness onset to hospitalization declined for those over 50 years (Fig 4A). Similarly, the time from symptom onset to death was inversely associated to age (Fig 4B). Reduced time-to-event periods in the elderly suggest a more severe manifestation of COVID-19 and a faster progression toward a fatal outcome compared to younger individuals.

### Demographics, comorbidity and clinical course

A total of 540 consecutive cases of acute COVID-19 disease were admitted at our hospital during the first wave of COVID-19 epidemic (March-April). The median age was 70 years (range 22–99) and 47% were female. The most common self-reported symptoms at admission were cough (66.5% of patients), dyspnea (61.6%), shortness of breath (38%), and fever > 38 ºC or 100.4 F (30.2%). Gastrointestinal symptoms such as diarrhea and anorexia were cited by 18.8% and 13.7% of patients respectively. Very elderly patients (>75 y) presented fewer mild symptoms on admission (cough, myalgia, headache) than the young (<60 y) and elderly (60–75), but more severe symptoms such as dyspnea, confusion, tachypnea, or lower oxygen saturation (Table 1). Radiological findings at admission also show significant differences according to age. The clinical features of the chest X-rays in very elderly patients include larger affectations and increased severity. Very elderly patients also presented more pleural effusion than elderly and young patients.

**Table 1:** Clinical characteristics and comorbidities according to age groups.

	<60	60–75	> = 75	N
	N = 158	N = 181	N = 198	
Cough	119 (75.8%)^A^	126 (69.6%)^A^	110 (55.8%)^B^	535
Artromyalgia	50 (31.6%)^A^	49 (27.1%)^A^	21 (10.7%)^B^	535
Headache	31 (19.7%)^A^	16 (8.84%)^B^	12 (6.19%)^B^	532
Dyspnoea	89 (56.7%)^A^	105 (58.0%)^A^	136 (69.0%)^B^	535
Confusion	4 (2.55%)^A^	11 (6.08%)^A^	49 (24.9%)^B^	535
Tachypnea	24 (15.3%)^A^	37 (20.7%)^A^	67 (34.5%)^B^	530
Crackles	75 (48.4%)^A^	97 (54.2%)^A,B^	119 (62.0%)^B^	526
Roncus	8 (5.16%)^A^	14 (7.82%)^A^	31 (16.1%)^B^	526
Oxygen saturation	96.0 [93.0;97.0]^A^	94.0 [90.0;96.0]^B^	93.0 [89.0;95.0]^C^	531
Chest X-ray severity	^A^	^A,B^	^B^	472
No affection	22 (15.0%)	27 (17.9%)	36 (20.7%)	
Mild	43 (29.3%)	28 (18.5%)	30 (17.2%)	
Moderate	69 (46.9%)	72 (47.7%)	64 (36.8%)	
Severe	13 (8.84%)	24 (15.9%)	44 (25.3%)	
HBP	34 (21.5%)^A^	98 (54.1%)^B^	149 (75.6%)^C^	536
Dyslipidemia	46 (29.1%)^A^	95 (52.5%)^B^	92 (46.9%)^B^	535
Diabetes	13 (8.23%)^A^	40 (22.1%)^B^	47 (23.9%)^B^	536
Dementia	0 (0.00%)^A^	5 (2.76%)^A^	60 (30.5%)^B^	536
IVD	11 (6.96%)^A^	38 (21.2%)^B^	75 (38.3%)^C^	533
CRD	25 (15.8%)^A^	43 (23.9%)^A,B^	53 (27.0%)^B^	534
IC	1 (0.63%)^A^	10 (5.52%)^B^	37 (18.8%)^C^	536
IRC	5 (3.16%)^A^	20 (11.1%)^B^	50 (25.4%)^C^	535
Charlson Comorbidity Index	1.00 [0.00;1.00]^A^	3.00 [2.00;5.00]^B^	5.00 [4.00;7.00]^C^	537

The prevalence of specific comorbidities among hospitalized individuals was 52.2% hypertension, 43.9% dyslipidemia, 22.8% ischemic cardiovascular disease, 22.6% chronic respiratory disease, 16.4% diabetes, 14.9% cancer, 13.9% chronic kidney disease, 11.9% dementia, and 8.8% heart failure. The average score on the Charlson Comorbidity Index was 3.8, corresponding to a 59% estimated 10-year survival. Both elderly and very-elderly groups had a higher prevalence of hypertension, dyslipidemia, diabetes, dementia, ischemic cardiovascular disease, chronic kidney disease and heart failure compared to those younger than 60 years. Charlson’s index (5 vs 1; p<0.001) was also higher in very-elderly patients than young ones. The presence and the number of comorbidities were a critical factor in predicting death in both elderly and very elderly patients but not in the <60 y age group (Fig 4C).

## Discussion

In this work, we describe a different clinical course and outcomes of COVID-19 in an age-dependent fashion. SARS-CoV-2 infection caused a severe and rapid progression in older patients who required faster hospitalization, presented more serious symptoms on admission, and had a worse clinical course. Hospitalized older individuals, even those without comorbidities, have an increased mortality risk directly associated with their age. Lastly, the existence of comorbidities dramatically increased the death rate in the elderly, especially in males.

From March to the end of November 2020, Spain endured more than 67,000 excess deaths compared to previous years [25], resulting in one of the countries with the highest number of deaths relative to its population (~47 mil). Risk stratification requires accurate and granular data, essential for identifying vulnerable individuals and for policy guidance [26]. Our data showed a spike in PCR-positive cases in Aragon among African farm laborers, just before the start of the second wave (S1 Fig). Had this been data either analyzed by public health authorities or made available in a timely manner for analysis by other researchers, the subsequent epidemic wave could probably have been neutralized or at least mitigated. Epidemiological surveillance systems must hence rely on real-time high-quality data [26]. Despite the proven usefulness of a rapid and comprehensive data analysis, the challenges associated with access to health data continue to occur in Spain at both the national and regional levels [27].

A chief implication of our data is that COVID-19 morbidity is crucially dependent on the age groups that are infected, which in turn reflects the population structure and aging of that population. Spain presents a relatively older population compared with other countries belonging to the Organization for Economic Co-operation and Development (OECD). This phenomenon is further amplified in Aragón (population: ~1.3 mill), one of the most aged Autonomous Communities within Spain with 42.7% being over 50 and a larger proportion of older persons in long-term care institutions. These factors might partially explain the high rates of serious disease and mortality compared to other territories. Nationwide seroepidemiological studies such as ENE-COVID [28] found a 6.8–9.5% seroprevalence of SARS-CoV-2 Aragon and more than 2,339 excess deaths compared to previous years by the end of November 2020 [25]. In this study we found that 85.5% of cases of COVID-19 in Aragón (Spain) resulted in mild illness, but 13.6% necessitated hospitalization, and 3.8% died. Adults over 60 years of age account for 96% of deaths caused by COVID-19. Compared to the first wave, we observed a sharp decrease in the hospitalization rate and CFR during the second and third waves. The reduction in morbidity and mortality occurred despite an increase in the incidence of the disease. This phenomenon could be explained, at least partially, by a decrease in serious side effects when certain drug regimens used initially for treatment and prophylaxis were discontinued (hydroxychloroquine, azithromycin, interferon, and lopinavir / ritonavir) as recommended by the Solidarity and Recovery trials [29, 30]. In addition, both the implementation of prophylactic heparin to reduce thrombotic complications [31] and the improvement in early ventilatory support in these patients has also played a key role in the decreased mortality observed during the second and third waves.

In this work, we found a similar incidence of COVID-19 in individuals between 10 and 79 years. Children under 10 years had a slightly lower incidence likely due to a reduced exposure in pre-school age. On the contrary, those over 80 years were disproportionately more affected, possibly because of the great transmission of the disease within the nursing homes [32]. COVID-19 showed a greater risk of severe disease with increasing age, a characteristic shared with the 2002–2004 SARS epidemic [33], influenza, and other respiratory virus [34]. Age as the most decisive factor related to mortality was rapidly identified since the beginning of the pandemic and confirmed in all subsequent published series and meta-analysis [35–37]. Aging has an important impact on the burden of diseases. Cardiovascular disease (CVD) affects disparately the elderly [38], and high burden of CVDs among COVID-19 patients is significantly associated with mortality and ICU admission [39]. Likewise, COVID-19 infection is associated with increased severity and mortality rates in patients with chronic obstructive pulmonary disease (COPD) [40], liver, and kidney diseases [41]. In addition, a decline in the immune function associated with age [11] can promote immunosenescence, hyperinflammation, hyperthrombosis, and cytokine storms, all of which are associated with COVID-19 vulnerability [42]. However, a deeper analysis is still needed in order to characterize the course of the disease in the elderly patients, who seems to be the most vulnerable population group. In this sense, this study adds some novel findings; we report a more severe manifestation of COVID-19 in the elderly and a faster progression toward a fatal outcome compared to younger individuals. Some previous studies, but not all [43], have also observed shorter hospital stays for deceased patients of all ages [44] and older [45], in line with the notion of a rapid progression of COVID-19 in patients with increased mortality.

On arrival to the emergency room, very elderly patients presented more severe radiological and respiratory symptoms. This increased severity may be explained as some older people cannot notice or clearly express early symptoms so they are diagnosed based on moderate respiratory symptoms. We also describe that patients’ fatality rate decreased after the first wave and remained stable during the two subsequent pandemic waves independently of the number of affected individuals as well as their median age. These findings highlight the age group of people over 80 years as the most vulnerable to SARS-CoV-2 infection in terms of mortality and further reinforce the necessity of smart targeted measures for this age group. We also found that the older the hospitalized patients were, the higher the risk even after controlling for relevant comorbidities. These data disagree with the theory that healthy older individuals can be at lower risk from COVID-19 than younger people with comorbidities.

The presence of severe symptoms that require hospitalization or cause death are much less common among young adults, as observed in previous reports [46–48]. Between March and October 2020, there was 1 deceased infant with severe underlying conditions and 227 hospital admissions in individuals between 0 and 29 years with positive PCR for COVID-19 in Aragon. To put these number in perspective, during the same period in 2015 there were 9 deaths and 62 hospitalizations due to traffic accidents in individuals younger than 29 years. Similar results have been recently shown using data from the Centers for Disease Control and Prevention (USA) in which young adults (<34 y) were more likely to die from heart disease, malignant neoplasms, traffic accidents, drug overdoses, suicides, or homicides than from COVID-19 during March trough October 2020 [49].

The Spanish Government, among many others, has enforced stay-at-home orders, curfews, mandatory isolation, closures on private business, restricted freedom of assembly, and set in place intrusive surveillance systems to monitor disease spread [50, 51]. All these extraordinary measures have indeed controlled the outbreaks in the short term. However, they seem to have little or no success in controlling the epidemic in the long run as indicated by the existence of various epidemic waves as soon as coercive measures were relaxed. On the other hand, older people were not considered priority health care targets, and a full implementation of specific preventive measures or comprehensive primary care were unfortunately lacking, with an excessive reliance on hospital care for the diseased [52]. We believe that overcoming this pandemic has to be based on an efficient strategy involving the entire population. However, we must keep in mind that the elderly are disproportionately affected by this disease. Therefore, preventive and therapeutic measures directed specifically towards older citizens will have a greater effect in minimizing the number of deaths. Vaccines against SARS-CoV-2 will offer the possibility of significantly reducing severe morbidity and mortality. However, they are unlikely to be immediately available in sufficient quantities to vaccinate the entire world population. We urge the authorities to prioritize the vaccination of older adults, specially those living in congregate or overcrowded facilities. While we do understand the need for stringent public health policies, we also believe that for such disruptive measures to be effective they must be tailored to those in the high-risk mortality category.

The main limitation of this study is that it is based on a single population and an external validation is hence warranted. This study has also several strengths: first, it accounts for nearly all cases of COVID-19 occurred in a precise region (Aragon, Spain). Second, its longitudinal nature which allowed to clearly identify changes across 3 different epidemic waves. Lastly, this study is based on the BIGAN repository; a high-quality and curated large database that provides real-life conditions.

Since the pandemic began, the prognostic value of different hematological, biochemical, inflammatory, and immunological biomarkers has been evaluated to predict the risk of severe or fatal COVID-19. The focus on the association of individual characteristics and disease progression may be obscuring the paramount importance of age as the major population determinant of mortality. Neglecting to take into account of the importance of the population context may jeopardize optimal resource allocation and clinical management as well as the prevention of serious complications. We believe that the best strategy for preventing overall morbidity and mortality in the initial phase of the vaccination campaigns is to directly protect persons most at risk of morbidity and mortality. The elevated incidence of COVID-19 and the situation of vulnerability of the elderly reveal the need for authorities to implement targeted prevention, detection, control and surveillance measures to guarantee the preferential access of the elderly to vaccines and other adequate preventive measures.

## Supporting information

S1 Fig Daily PCR-confirmed COVID-19 cases (top). Country of birth (%) of all PCR-tested individuals, including both positive and negative cases (middle). Longitudinal variations in the percentage of positive cases according to the country of birth with respect to the total number of daily positives (bottom). Different superscript letters indicate statistically significant differences between variables. HBP; High blood pressure. IVD; Ischemic cardiovascular disease: acute myocardial infarction, angina pectoris, ischemic stroke, and peripheral arterial disease. CRD: Chronic respiratory disease: COPD, bronchitis, asthma, and apnea-hypopnea syndrome. IC: Cardiac insufficiency (heart failure) IRC: Chronic kidney disease.
(TIF)
