# Supplementary figures and images for: Age-related mortality in 61,993 confirmed COVID-19 cases over three epidemic waves in Aragon, Spain. Implications for vaccination programmes
Source: PLoS One. 2021 Dec 9;16(12):e0261061. doi: 10.1371/journal.pone.0261061 (PMC8659616; doi:10.1371/journal.pone.0261061)

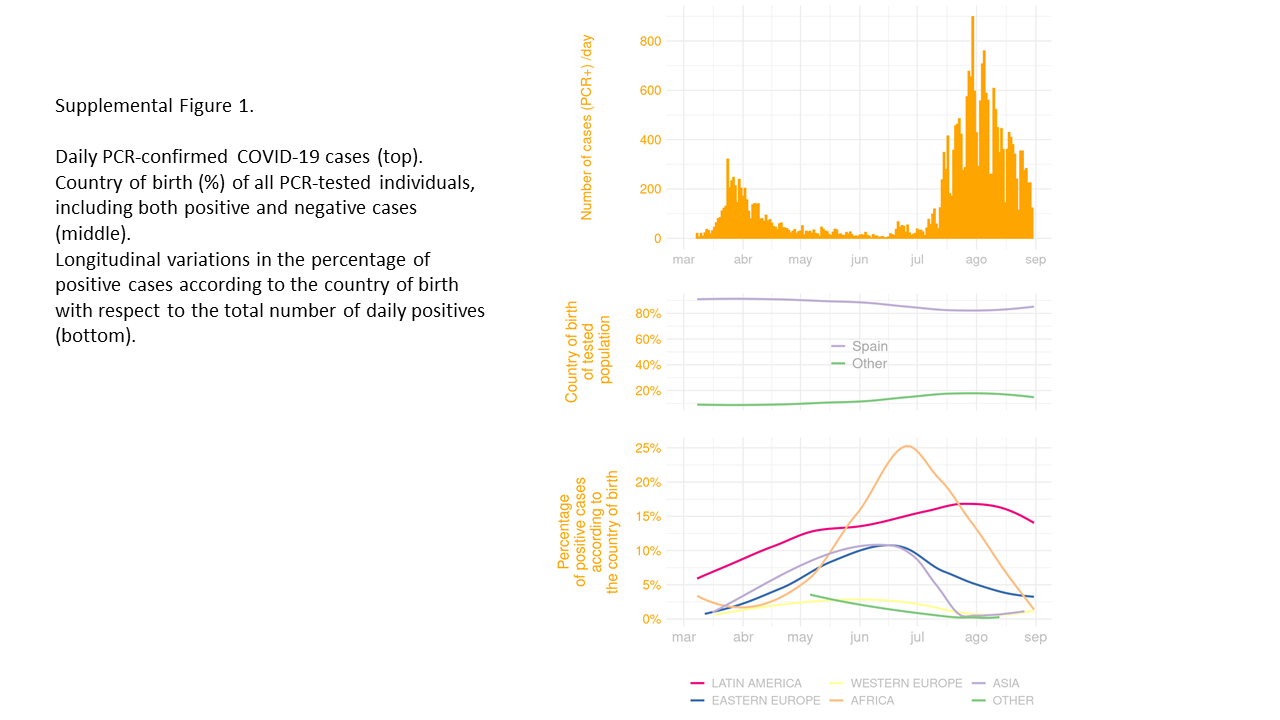

Supplement: S1 Fig — Country of birth (%) of all PCR-tested individuals, including both positive and negative cases (middle). Longitudinal variations in the percentage of positive cases according to the country of birth with respect to the total number of daily positives (bottom). Different superscript letters indicate statistically significant differences between variables. HBP; High blood pressure. IVD; Ischemic cardiovascular disease: acute myocardial infarction, angina pectoris, ischemic stroke, and peripheral arterial disease. CRD: Chronic respiratory disease: COPD, bronchitis, asthma, and apnea-hypopnea syndrome. IC: Cardiac insufficiency (heart failure) IRC: Chronic kidney disease. (TIF) [file pone.0261061.s001.tif]
